# EZH2 promotes cell migration and invasion but not alters cell proliferation by suppressing E-cadherin, partly through association with MALAT-1 in pancreatic cancer

**DOI:** 10.18632/oncotarget.7156

**Published:** 2016-02-03

**Authors:** Ting Han, Feng Jiao, Hai Hu, Cuncun Yuan, Lei Wang, Zi-Liang Jin, Wei-feng Song, Li-Wei Wang

**Affiliations:** ^1^ Department of Medical Oncology and Pancreatic Cancer Center, Shanghai General Hospital, Shanghai Jiao Tong University School of Medicine, Shanghai 201620, China; ^2^ Shanghai Key Laboratory of Pancreatic Diseases, Shanghai 201620, China; ^3^ Department of Pathology, Shanghai General Hospital, Shanghai Jiao Tong University School of Medicine, Shanghai 201620, China

**Keywords:** pancreatic cancer, enhancer of zeste homolog 2, metastasis associated lung adenocarcinoma transcript 1, long non-coding RNA, cell migration

## Abstract

Enhancer of zeste homolog 2 (EZH2) is an essential component of the polycomb repressive complex 2 (PRC2), which is required for epigenetic silencing of target genes, including those affecting cancer progression. Its role in pancreatic cancer remains to be clarified; therefore, we investigated the effects of aberrantly expressed EZH2 on pancreatic cancer. We found that EZH2 expression is up-regulated in pancreatic cancer tissues and positively correlated with lymph node metastasis and advanced clinical stage in pancreatic cancer patients. EZH2 knockdown in pancreatic cancer cell lines inhibited cell migration and invasion, but did not alter cell proliferation. Silencing of EZH2 also increased E-cadherin expression *in vitro*, and E-cadherin expression was inversely correlated with EZH2 expression in pancreatic cancer tissue samples. Patients with high EZH2 and low E-cadherin expression had the worst prognosis. RIP and ChIP assays suggest that EZH2 is recruited to the E-cadherin promoter by the long non-coding RNA, MALAT-1 (metastasis associated in lung adenocarcinoma transcript 1), where it represses E-cadherin expression. Our results show that EZH2-based therapies may be an option for the treatment of pancreatic cancer.

## INTRODUCTION

It is estimated that there are approximately 48,960 new cases of pancreatic cancer diagnosed annually around the world [1]. With 5-year survival rates of less than 5%, this extremely malignant tumor type exhibits rapid progression with no obvious symptoms, so it is often at an advanced stage when diagnosed. This makes it difficult to identify early diagnostic markers and design drugs to target pancreatic cancer. While gene-based therapies have recently been explored, there has been more interest in the critical role of epigenetic modifications for controlling the activity of genes involved in lineage specification [2–4], differentiation [5–7], and tissue renewal [8–10].

Enhancer of zeste homolog 2 (EZH2) is a catalytic subunit of polycomb repressive complex 2 (PRC2), which represses genes involved in tumorigenesis (e.g., hMLH1, ARHI and RASSF1A in ovarian cancer) via methylation of lysine 27 of histone 3 (H3K27) [11]. EZH2 is over-expressed in a wide range of tumors, including breast [12], prostate [13], bladder [14], lung [15] and colorectal cancer [16]. In addition, it was recently reported that two other histone marks, H2AK119Ub1 and H3K27Me3, cooperate and are associated with clinical prognosis [17]. EZH2 up-regulation has been implicated in acute pancreatitis, where it promotes tissue repair through regenerative proliferation of progenitor cells, resulting in impaired pancreatic regeneration and accelerating Kras-driven neoplasia [18].

Deregulation of long non-coding RNAs (lncRNAs) has also been linked to various cancers. LncRNAs act as signals, decoys, guides, and scaffolds, responsible for emerging tumor archetypes [19]. Previously, our group has focused on the lncRNA MALAT-1 (metastasis associated in lung adenocarcinoma transcript 1), which is an oncogenic lncRNA involved in the malignancy of pancreatic cancer via induction of G2/M cell cycle arrest, promotion of cell apoptosis, suppression of epithelial-mesenchymal transition (EMT) and reduction of cancer stem-like properties [20, 21]. However, the role of EZH2 in pancreatic cancer and its interaction with MALAT-1 remain unclear. In this study, we investigated EZH2 expression in pancreatic cancer, assessed its biological functions, and performed an initial analysis of its molecular mechanisms of action.

## RESULTS

### Aberrant over-expression of EZH2 in human pancreatic cancer tissues

**Figure 1:**
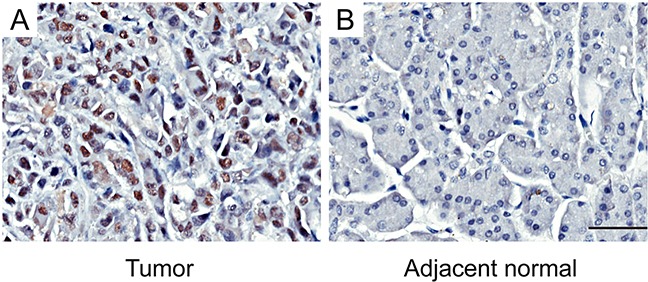
Immunohistochemical staining for EZH2 expression in pancreatic cancer tissues and adjacent normal tissuesRepresentative figures of EZH2 expression in pancreatic cancer tissues and adjacent normal tissues **A.** EZH2 is highly expressed in tumor tissues. **B.** The expression of EZH2 in adjacent normal tissues is negative. The scales represent 50μm.

Tissue microarrays (TMAs) were used to evaluate the EZH2 expression in pancreatic cancer. As shown in Figure 1, EZH2 protein was localized in the cell nucleus. Of 84 tumor tissue samples, 42 (50%) pancreatic cancers had high EZH2 expression, whereas only 7 (8.3%) had high expression in adjacent non-tumor tissues. These results suggest that EZH2 expression is up regulated in pancreatic cancer compared to non-tumor tissues (*P*<0.05; Figure 1, Table 1).

**Table 1:** Protein expression of E-cadherin and EZH2 in pancreatic cancer tissues and adjacent normal tissues

Tissue sample	No. of patients	E-cadherin		*P*-value	EZH2		*P*-value
		Low (%)	High (%)		Low (%)	High (%)	
Tumor	84	31(36.9)	53(63.1)	0.001*	42(50.0)	42(50.0)	<0.001*
Non-cancerous tissues	84	12(14.3)	72(85.7)		77(91.7)	7(8.3)	

EZH2 and E-cadherin expression were measured in tumor and non-cancerous tissues. EZH2 was higher in tumor tissues whereas E-cadherin was higher in non-cancerous tissues. Data were analyzed using the Chi-squared test.

* *P* < 0.05 indicates statistical significance.

### Correlation between EZH2 over-expression and clinico-pathological characteristics of pancreatic cancer

**Figure 2:**
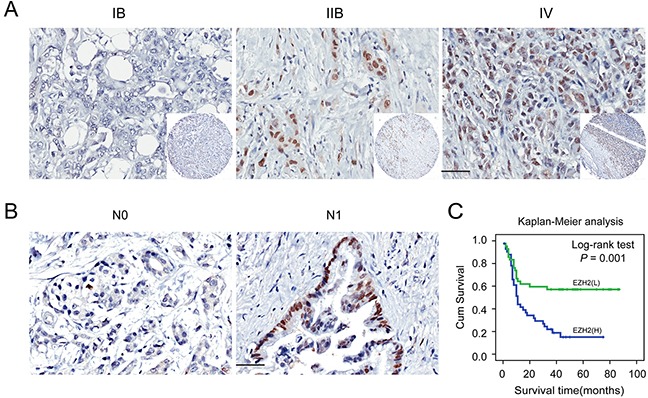
Immunohistochemical staining for EZH2 protein expression with or without lymph nodes metastasis and with different clinical stages of pancreatic cancer Representative figures of EZH2 expression in patient with or without lymph node metastasis and with different clinical stages and the prognosis the patients. **A.** EZH2 expression is positively correlated with advanced tumor stage. **B.** EZH2 expression is positively correlated with lymph node metastasis. **C.** Patients with higher expression of EZH2 possessed a worse prognosis. The scales represent 50μm. “L” represents low, “H” represents high.

We next correlated EZH2 expression with clinico-pathological features in pancreatic cancer patients. EZH2 was positively correlated with clinical stage (*P* = 0.015; Figure 2A; Table 2) and lymph node metastasis (*P* = 0.044; Figure 2B; Table 2). Other clinico-pathological features such as gender and age were not correlated with EZH2 expression (Table 2). These data suggest that EZH2 may be involved in pancreatic cancer progression.

**Table 2:** Associations between E-cadherin, EZH2 protein expression and clinico-pathological characteristics in pancreatic cancer

Clinico-pathological parameters	No. of patients	E-cadherin		*P*-value	EZH2		*P*-value
		Low (%)	High (%)		Low (%)	High (%)	
Cases	84	31(36.9)	53(63.1)		42(50.0)	42(50.0)	
Age(years)							
≤60	39	17(43.6)	22(56.4)	0.237^a^	16(41.0)	23(59.0)	0.126^a^
>60	45	14(31.1)	31(68.9)		26(57.8)	19(42.2)	
Gender							
Male	51	21(41.2)	30(58.8)	0.313^a^	28(54.9)	23(45.1)	0.264^a^
Female	33	10(30.3)	23(69.7)		14(42.4)	19(57.6)	
Tumor location							
Head,neck	56	24(42.9)	32(57.1)	0.110^a^	26(46.4)	30(53.6)	0.355^a^
Body,tail	28	7(25.0)	21(75.0)		16(57.1)	12(42.9)	
Tumor size(cm)							
≤3	25	9(36.0)	16(64.0)	0.911^a^	10(40.0)	15(60.0)	0.233^a^
>3	59	22(37.3)	37(62.7)		32(54.2)	27(45.8)	
Tumor differentiation							
Well, moderate	57	21(36.8)	36(63.2)	0.986^a^	30(52.6)	27(47.4)	0.483^a^
Poor	27	10(37.0)	17(63.0)		12(44.4)	15(55.6)	
Invasion depth							
T1+T2	71	27(38.0)	44(62.0)	0.618^a^	36(50.7)	35(49.3)	0.763^a^
T3+T4	13	4(30.8)	9(69.2)		6(46.2)	7(53.8)	
Lymph nodes metastasis							
N0(negative)	51	14(27.5)	37(72.5)	0.026^a^*	30(58.8)	21(41.2)	0.044^a^*
N1(positive)	33	17(51.5)	16(48.5)		12(36.4)	21(63.6)	
Distant metastasis							
M0(absent)	82	29(35.4)	53(64.6)	0.133^b^	42(51.2)	40(48.8)	0.494^b^
M1(present)	2	2(100)	0(0)		0(0)	2(100)	
Clinical stage							
Early stages (≤IIa)	49	13(26.5)	36(73.5)	0.020^a^*	30(61.2)	19(38.8)	0.015^a^*
Advanced stages (>IIa)	35	18(51.4)	17(48.6)		12(34.3)	23(65.7)	
Nervous invasion							
negative	51	20(39.2)	31(60.8)	0.585^a^	26(51.0)	25(49.0)	0.823^a^
positive	33	11(33.3)	22(66.7)		16(48.5)	17(51.5)	

a Chi-square test.

b Fisher's exact test.

* *P*<0.05 indicates a significant association among the variables.

### High EZH2 expression is associated with poor clinical outcomes in pancreatic cancer patients

To further explore the relationship between EZH2 expression and patient prognosis, we conducted a Kaplan-Meier analysis. Patients were subdivided according to EZH2 IHC scores. High EZH2 expression was associated with an especially poor prognosis for pancreatic cancer patients (*P*<0.05; Figure 2C). Univariate survival analysis revealed that, in addition to tumor differentiation, lymph node metastasis, and tumor stage, high EZH2 expression predicted poor prognosis (*P* < 0.05). COX regression model analysis showed that high EZH2 expression and lymph node metastasis were correlated with overall survival (OS) (HR = 2.2041, *P* = 0.011; Table 3). Together, these findings demonstrated that EZH2 expression could be an independent prognostic marker for pancreatic cancer.

**Table 3:** Univariate and multivariate Cox regression of prognostic factors for overall survival in pancreatic cancer

Clinico-pathological parameters	Univariate analysis			Multivariate analysis		
	HR	95% CI	*P*-value	HR	95% CI	*P*-value
EZH2						
Low	1			1		
High	2.556	1.433-4.559	0.001*	2.204	1.199-4.049	0.011*
E-cadherin						
Low	1					
High	0.479	0.277-0.830	0.009*			
Age(years)						
≤60	1					
>60	0.922	0.535-1.588	0.922			
Gender						
Male	1					
Female	0.573	0.314-1.046	0.070			
Tumor location						
Head,neck	1					
Body,tail	1.227	0.698-2.159	0.477			
Tumor size(cm)						
≤3	1					
>3	0.843	0.472-1.507	0.565			
Tumor differentiation						
Well, moderate	1			1		
Poor	1.992	1.134-3.500	0.017*	2.235	1.268-3.942	0.005*
Invasion depth						
T1+T2	1					
T3+T4	1.005	0.472-2.137	0.991			
Lymph nodes metastasis						
N0(negative)	1			1		
N1(positive)	2.151	1.243-3.723	0.006*	1.881	1.056-3.349	0.032*
Distant metastasis						
Absent	1					
Present	2.443	0.591-10.101	0.217			
Clinical stage						
Early stages (≤IIa)	1					
Advanced stages (>IIa)	2.338	1.348-4.055	0.002*			
Nervous invasion						
negative	1					
positive	1.209	0.699-2.092	0.498			

HR: hazard ratio; 95% CI: 95% confidence interval

* *P* <0.05 indicates that 95% CI of HR is not including 1.

### EZH2 knockdown does not decrease pancreatic cancer cell proliferation *in vitro*

**Figure 3:**
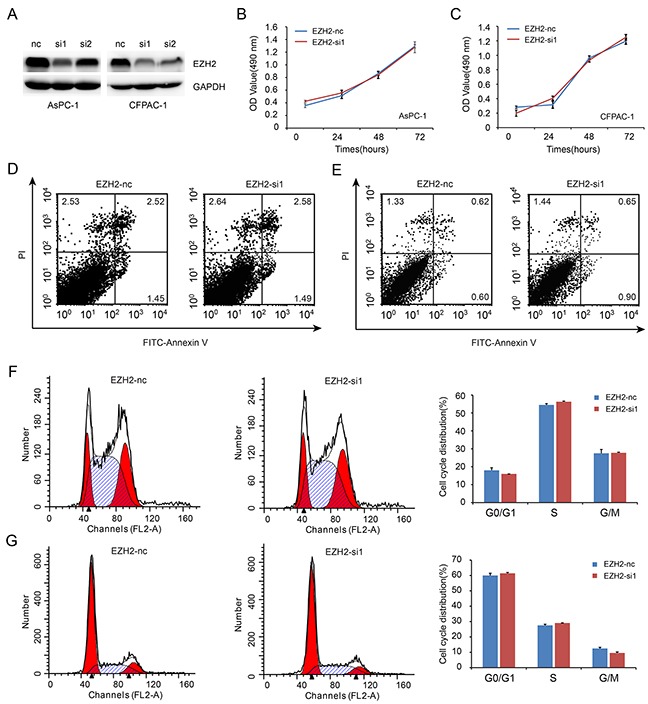
EZH2 knockdown in pancreatic cancer cells shows no effect on cell proliferation **A.** We designed siRNA fragment specifically targeted EZH2, and chose the higher knockdown efficiency fragment to perform the following experiment. **B, C.** EZH2 knockdown do not alter cell proliferation. AsPC-1(B), CFPAC-1 (C). **D, E.** Flow cytometric analysis showed that the ratio of cell apoptosis do not altered after EZH2 knockdown. AsPC-1(D), CFPAC-1(E). **F, G.** Flow cytometry analysis showed that the ratio of cell cycle do not altered after EZH2 knockdown. AsPC-1(F), CFPAC-1(G).

To investigate the role of EZH2 in pancreatic cancer cell proliferation, we decreased EZH2 expression in AsPC-1 and CFPAC-1 cell lines using EZH2-siRNA. As shown in Figure 3A, EZH2-si1 achieved the greatest efficacy in silencing EZH2 expression. Thus, EZH2-si1 was used to examine the effects of decreased EZH2 expression on pancreatic cancer cell proliferation. We found no difference between growth curves of EZH2 knockdown cells when compared with controls (Figure 3B, 3C). Then, we performed flow cytometric analysis to assess whether cell cycle and apoptosis were altered. There were no differences in cell cycle stage or amount of apoptosis after EZH2 knockdown (Figure 3D, 3E, 3F, 3G).

### EZH2 promotes pancreatic cancer cell migration and invasion *in vitro* through repression of E-cadherin

**Figure 4:**
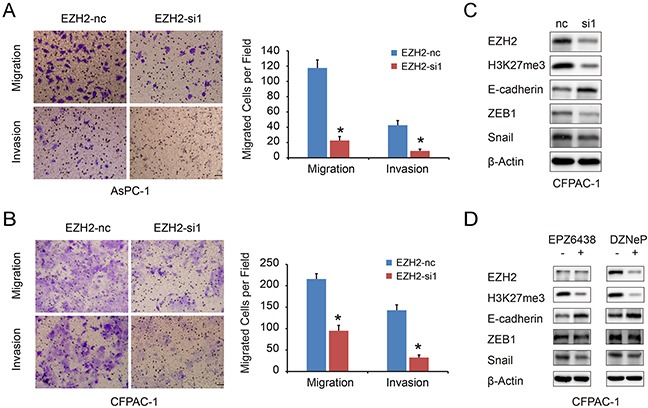
EZH2 knockdown in pancreatic cancer cells inhibits cell migration and invasion **A, B.** The migration and invasion ability of pancreatic cancer cell lines AsPC-1(A), CFPAC-1(B) with EZH2 knockdown. The scales represent 50μm. **C, D.** we examined the E-cadherin, ZEB1 and Snail expressions after using EZH2 RNAi, DZNeP and EPZ-6438. “*”represent *P*<0.05 when compared with control group.

We used a transwell migration assay to examine the effect of EZH2 on pancreatic cancer cell migration and invasion. EZH2-si1 knockdown reduced the number of migrating cells as compared to EZH2-nc cells, indicating decreased migratory and invasion abilities following EZH2 knockdown (Figure 4A, 4B). Previous reports found that EZH2 causes transcriptional silencing of the tumor suppressor gene E-cadherin [22], and that lower expression of E-cadherin in pancreatic cancer is correlated with increased migration and invasion [23]. Therefore, repression of EZH2 could restore E-cadherin expression [24, 25]. We found that silencing EZH2 increased E-cadherin expression in pancreatic cancer (Figure 4C). In addition, EZH2 selective inhibitors EPZ-6438 and DZNeP increased E-cadherin expression in pancreatic cancer cell lines (Figure 4D). Pharmacologically, DZNeP inhibited both EZH2 and H3k27me3 expression while EPZ-6438 only inhibited H3K27me3 [26, 27]. Besides, the EMT-related transcription factors, ZEB1 and Snail, were also decreased after EZH2 knockdown (Figure 4D, Supplementary Figure S1A, S1B). These results indicated that EZH2-mediated H3K27 tri-methylation may cause repression of E-cadherin in pancreatic cancer, and that ZEB1 and Snail might also be involved in this process.

### EZH2 expression is inversely correlated with E-cadherin expression in pancreatic cancer tissues

To further confirm the association of EZH2 and E-cadherin in pancreatic cancer, we assessed E-cadherin expression in the TMAs data. EZH2 expression was inversely correlated with E-cadherin expression (*P*<0.001; Table 4). Kaplan-Meier analysis indicated that high E-cadherin expression in tumor samples favored good clinic outcomes (Supplementary Figure S2A). Notably, a combinatorial pattern of high EZH2 expression and low E-cadherin predicted the worst clinical prognosis in pancreatic cancer (Supplementary Figure S2B).

**Table 4:** Correlation analysis between EZH2 and E-cadherin protein expression in pancreatic cancer

Tumor tissue sample	E-cadherin		Correlation coefficient	*P*-value
	Low	High		
EZH2 Low	7	35	−0.419	<0.001*
EZH2 High	24	18		

* *P* < 0.05 indicates statistical significance.

### EZH2 inhibits E-cadherin expression partly through association with MALAT-1

**Figure 5:**
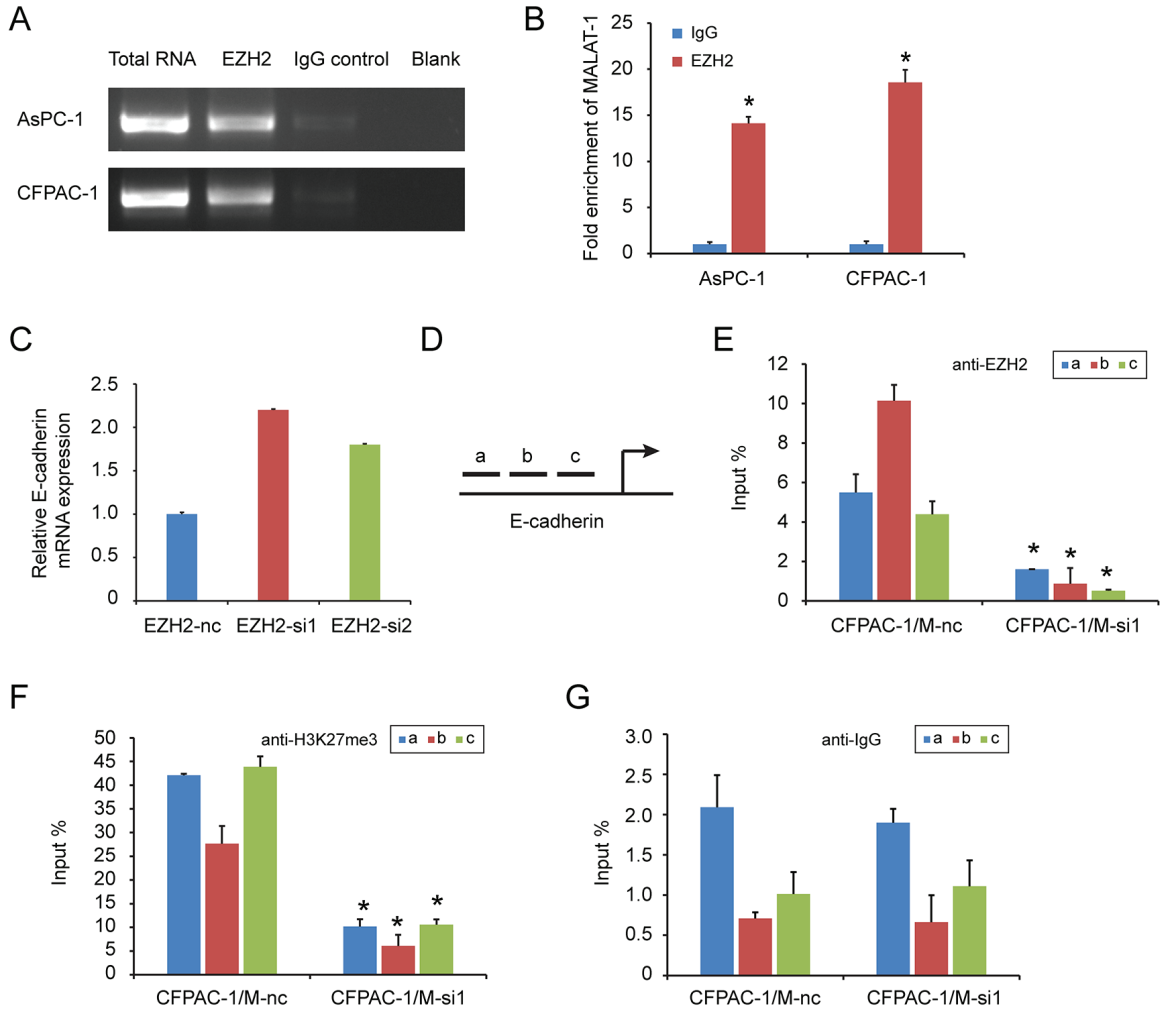
EZH2 is recruited by MALAT-1 to binding to E-cadherin promoter, suppressing E-cadherin expression RIP experiments were performed using the EZH2 antibody to immunoprecipitate RNA and a primer to detect MALAT-1 RNA. **A, B.** Compared to IgG, anti-EZH2 antibody group is rich in MALAT-1. Chromatin Immunoprecipitation (ChIP) assays are conducted on E-cadherin promoter region (primer set a–c) using the indicated antibodies. Enrichment is determined relative to input controls. **C.** EZH2 interference increased E-cadherin mRNA level. **D.** We design three pairs of primers (a, b, c) targeting E-cadherin promoter region. **E, F, G.** Our targeted antibody EZH2(C), H3K27me3 (D) and negative control IgG (E), were used for ChIP experiment. Inference of MALAT-1 reduced the recruitment of EZH2 in E-cadherin promoter region. “*” represent *P*<0.05 when compared with control group.

LncRNA can regulate gene transcription and expression through histone modifications, and EZH2 contains a potential lncRNA-binding site [28]. We previously found that a lncRNA, MALAT-1, suppresses E-cadherin expression [21]. In this study, there was a positive correlation of EZH2 and MALAT-1 expression in pancreatic cancer tissues (Table 5). MALAT-1 was also inversely correlated with E-cadherin expression (Table 6). And knockdown of MALAT-1 upregulated E-cadherin mRNA expression in pancreatic cancer cell lines (Supplementary Figure S3). Therefore, we hypothesized that EZH2 might be recruited by MALAT-1 to synergistically repress E-cadherin. To test this hypothesis, we first asked whether MALAT-1 bound to EZH2 using a RNA Immunoprecipitation (RIP) assay. As shown in Figure 5A and 5B, there was an average of 14- and 18- fold enrichment for MALAT-1 in the AsPC-1 and CFPAC-1 cells over-expressing EZH2, respectively, as compared to the IgG group. These results suggest that MALAT-1 is physically associated with the EZH2, and silencing EZH2 could increase E-cadherin transcription (Figure 5C).

**Table 5:** Correlation analysis between MALAT-1 and EZH2 mRNA expression in paraffin specimens of pancreatic cancer

Tumor tissue sample	MALAT-1		Correlation coefficient	*P*-value
	Low	High		
EZH2(Low)	13	4	0.542	0.001*
EZH2(High)	4	14		

* *P* < 0.05 indicates statistical significance.

**Table 6:** Correlation analysis between MALAT-1 and E-cadherin mRNA expression in paraffin specimens of pancreatic cancer

Tumor tissue sample	MALAT-1 expression		Correlation coefficient	*P*-value
	Low	High		
E-cadherin(Low)	5	12	−0.373	0.028*
E-cadherin(High)	12	6		

* *P* < 0.05 indicates statistical significance.

Next, we used a chromatin immunoprecipitation (ChIP) to examine whether EZH2 could be recruited to the E-cadherin promoter by MALAT-1. We designed three primer pairs targeting the E-cadherin promoter (Figure 5D), and assessed the enrichment of EZH2 after MALAT-1 interference. Depletion of MALAT-1 caused a reduction in EZH2 binding to the E-cadherin promoter (Figure 5E, 5G). Momparler and colleagues [29] found that EZH2 suppresses gene expression through H3K27me3. We also performed ChIP using an H3K27me3 antibody, and found decreased enrichment of H3K27me3 at the E-cadherin promoter when compared to IgG antibody (Figure 5F, 5G). Taken together, these data suggest that EZH2 is recruited by MALAT-1 to the promoter of E-cadherin, and that together they repress E-cadherin expression.

## DISCUSSION

EZH2 is oncogenic in a wide variety of cancer types, functioning predominately as a transcriptional repressor that silences tumor suppressor gene targets [30]. There are physical and functional links between EZH2 and both DNA methyltransferases (DNMTs) [31] and histone deacetylases (HDACs) [32, 33]. In addition, EZH2 can function as a gene activator [34–36]. For example, EZH2 up-regulates oncogenes in metastatic prostate cancer and may be a valuable prognostic indicator of patient outcomes [37, 38]. Many cancer cell types exhibit aberrant EZH2 expression, and EZH2 expression is highly correlated with tumor invasiveness in breast cancer [39, 40].

We initially examined EZH2 expression in pancreatic cancer tissues and corresponding adjacent non-cancerous tissues, and confirmed strong EZH2 expression in tumor tissues. Moreover, we found that increased EZH2 expression was an independent negatively predictive factor for pancreatic cancer patients' clinical outcomes. We next began to explore the molecular functions of EZH2 in pancreatic cancer cell lines. We observed that EZH2 knockdown inhibited cell migration and invasion, but did not alter cell proliferation, cell cycle, or apoptosis.

Liu et al. [41] found that EZH2 promoted tumor cell migration and invasion via epigenetic repression of E-cadherin in renal cell carcinoma. Another study, by Wang and colleagues [42], found that EZH2-mediated E-cadherin repression promoted metastasis in tongue squamous cell carcinoma. Low expression of E-cadherin in tumors is correlated with tumor cell invasion and metastasis. Yang et al. [43] previously showed that down-regulation of E-cadherin was associated tumor progression and was an important predictive factor for non-small cell lung cancer. Weak expression of E-cadherin might be due to hypermethylation of the E-cadherin promoter [44]. We found that silencing EZH2 using RNAi or selective EZH2 inhibitors increased E-cadherin expression. We also examined transcription factor expression and found that ZEB1 and Snail expression were decreased after EZH2 silencing in pancreatic cancer cell lines. Moreover, EZH2 expression correlated inversely with E-cadherin expression in pancreatic cancer tissue samples, and overall survival among patients with high EZH2 expression and low E-cadherin expression was the shortest. Taken together, our results indicated that EZH2 promotes pancreatic cancer migration and invasion, possibly through the repression of E-cadherin expression, leading to a poor prognosis in pancreatic cancer patients.

**Figure 6:**
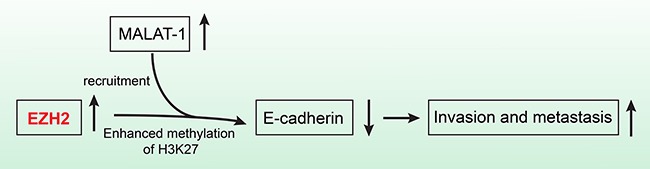
The schematic illustration of the EZH2 mechanism in pancreatic cancer EZH2 recruitment by MALAT-1 and subsequent involvement of the complex in the repression of E-cadherin, promotes pancreatic cancer migration and invasion.

EZH2 binds to the lncRNAs EBIC [45] and HOTAIR [46]. In addition, epigenetic silencing of the lncRNA SPRY4 occurs in non-small cell lung cancer cells through direct transcriptional repression mediated by EZH2 [47]. We previously showed that the lncRNA MALAT-1 promoted proliferation, invasion, and metastasis in pancreatic cancer [21]. In addition, we found a positive correlation between EZH2 and MALAT-1 expression in pancreatic cancer tissues. MALAT-1 was also inversely correlated with E-cadherin expression. And knockdown of MALAT-1 upregulated E-cadherin mRNA expression in pancreatic cancer cell lines. We therefore hypothesized that EZH2 regulated E-cadherin through an association with MALAT-1. Our RIP results indicated that EZH2 bound to MALAT-1. Furthermore, our ChIP results showed that MALAT-1 interference reduces EZH2 recruitment to the E-cadherin promoter. A recent study of aggressive renal cell carcinoma [48] corroborates our findings. We now hypothesize that EZH2 is recruited by MALAT-1 and increases H3K27me3 at the E-cadherin promoter, thereby suppressing E-cadherin expression and leading to a malignant phenotype of enhanced migration and invasion (Figure 6).

In conclusion, our data indicate that EZH2 recruitment by MALAT-1 and subsequent involvement of the complex in the repression of E-cadherin, promotes pancreatic cancer migration and invasion. EZH2 could therefore be an independent prognostic factor for pancreatic cancer patients as well as a novel and efficient molecular target for pancreatic cancer therapies.

## MATERIALS AND METHODS

### Tissue microarrays and immunohistochemistry

Tissue microarrays (TMAs) were purchased from ShGnghGi Outdo Biotech Company (China), containing 90 pancreatic cancer tissues and corresponding non-tumor tissues. The TMAs contained well-documented clinicopathological information, including patients' age, sex, tumor size and location, pathological grade, peri-neural invasion, lymph node metastasis, tumor stage and follow-up data (ended in December, 2011). Six patients were excluded due to lack of completed clinical and follow-up data. In total, 84 patients were included, 51 males and 33 females, with a median age of 62 years old (ranging from 38 to 85 years old). The overall survival time ranged from 0 to 87 months, with a median time of 15 months. Detailed information can be found in Table 2.

Samples were deparaffinized in xylene and rehydrated in a series of graded alcohol. Endogenous peroxidases were blocked by 3% H_2_O_2_, and antigen retrieval was completed after heating in citrate buffer. The sections were incubated with a rabbit polyclonal antibody against EZH2 (dilution 1:20; CST, Cambridge, UK) or E-cadherin(dilution 1:100; BD) at 4°C overnight and then with horseradish peroxidase (HRP) (Gene Tech GTVision III Detection Kit, Shanghai, China) at room temperature for 40 min. Following 3 washes with PBS, the signal was detected with 3, 3′-diaminobenzidine (DAB) solution. Breast and lung cancer tissue samples were used as a positive control. (Supplementary Figure S4)

### Scoring of immunohistochemistry

A double-blind method was used to analyze immunohistochemistry results and the evaluation was carried out independently by two investigators, without access to the patients' clinical and pathological features. Five visual fields from different areas of each specimen were chosen at random for the immunohistochemistry evaluation. EZH2 and E-cadherin expression was scored according to staining intensity and the percentage of positive cells as previously described [49]. The percentage of positive cells was scored as follows: 0, no positive cells; 1, ≤10% positive cells; 2, 10–50% positive cells; 3, >50% positive cells. Staining intensity was scored as follows: 0, no staining; 1, faint staining; 2, moderate staining; 3, dark staining. Comprehensive score = staining percentage × intensity. E-cadherin expression was classified as follows: ≤4 low expression, >4 high expression: EZH2 expression: < 2 low expression, ≥ 2 high expression.

### Cell lines, human samples, and reagents

Pancreatic cancer cells lines CFPAC-1, SW1990, AsPC-1 were all obtained from the Chinese Academy of Sciences Cell Bank (Shanghai, China). AsPC-1 and CFPAC-1cells were cultured in RPMI 1640 (Gibco, USA) supplemented with 10% fetal bovine serum (FBS), grown in 5% CO_2_ saturated humidity at 37°C and sub-cultured by harvesting with trypsin-EDTA. SW1990 cells were cultured in L-15 medium (Gibco) supplemented with 10% FBS, and grown in room temperature air.

Paraffin embedded tissue samples were collected from the surgery department, Shanghai General Hospital. Written informed consent was obtained from all subjects, and the study was approved and supervised by the Ethics Committee of the Shanghai General Hospital, Shanghai Jiao Tong University School of Medicine.

Selective EZH2 inhibitors, EPZ-6438 and DZNeP (Selleck), were used to examine differential expression of proteins after EZH2 knockdown.

### RNA interference (RNAi)

Pancreatic cell lines AsPC-1 and CFPAC-1 were plated in 6-wells plates, and transfected with 3μl EZH2-RNAi in the presence of 4μl RNAimax according to manufactures' instructions. Two different 21-nucleotide duplex siRNAs for EZH2 and one negative control siRNA were synthesized by Genepharm Technologies (Shanghai, China). The two EZH2 RNAi sequences were as follows: EZH2-si 1, 5′-GGAUGGUACUUUCAUUGAATT-3′; EZH2-si 2, 5′-CGGCUUCCCAAUAACAGUATT-3′, and the scramble sequence: 5′-UUCUCCGAACGUGUC ACGUTT-3′. Gene silencing effects were confirmed by Western blot and Real-time-quantitative polymerase chain reaction (RT-qPCR) analysis at 48 hours post-transfection.

### RNA isolation and RT-qPCR analysis

Total RNA was isolated from the cultured cells by using miniBEST universal RNA extraction kit (Takara Bio, Inc.) and tissue samples using a miRNeasy FFPE Kit (QIAGEN) according to the manufacturer's instructions. Reverse transcription and RT-qPCR kits (Takara Bio, Inc.) were used to evaluate expression of EZH2 and E-cadherin. GAPDH expression was used to normalize for variance. The PCR Primers pairs used for each genes were:

EZH2 forward, 5′-CCGCAAGGGTAACAAAAT-3′;
EZH2 reverse, 5′-GGTAGCAGATGTCAAGGGA-3′;
E-cadherin, forward, 5′-TGTCCGCCCCGACTTGTCTCTC-3′;
E-cadherin reverse, 5′-GTCCTCTGGCCCCAGCCTCTCT-3′;
GAPDH, forward, 5′-CCCCGCTACTCCTCCTCCTAAG-3′;
GAPDH reverse, 5′-TCCACGACCAGTTGTCCATTCC-3′;
MALAT-1 forward, 5′-GAATTGCGTCATTTAAAGCCTAGTT-3′;
MALAT-1 reverse, 5′-GTTTCATCCTACCACTCCCAATTAAT-3′.

Relative mRNA expression of each gene was calculated with the comparative threshold cycle (Ct) (2^−ΔΔCt^) method.

### Western blot analysis

Cells were lysed in RIPA lysis buffer and the protein concentration was determined by a standard Bradford assay (Beyotime). An equal amount of protein (10μg) from each cell line was subjected to Western blot analysis. Total proteins were fractionated using SDS-PAGE and transferred onto a polyvinylidene fluoride membrane. The membranes were blocked in 3% bovine serum albumin in TBST buffer containing 0.1% Tween-20 and then incubated with the indicated primary antibodies at 4°C overnight. Appropriate secondary antibodies were incubated at room temperature for 1 h and detected using the enhanced chemiluminescence detection system. The data were adjusted against loading control using β-Actin or GAPDH. The probing antibodies used for western blot analyses were: anti-E-cadherin (BD Biosciences, Bedford, MA, USA); anti-H3K27me3, anti-EZH2, anti-ZEB1, anti-Snail, anti-GAPDH (Cell Signaling Technology); and anti-β-Actin (Santa Cruz Biotechnology, Inc., Santa Cruz, CA, USA).

### Cell proliferation assay

The proliferation assay was performed using the SRB method. Cell were seeded in 96-well microtiter tissue culture plates and cultured for 24 h. AsPC-1 and CFPAC-1 cells were transfected with EZH2-nc and EZH2-si1 and cultured for 24, 48, 72h, respectively. At the end of the treatment, cells were fixed with 10% w/v of trichloroacetic acid (100 μl) for 1 h at 4°C. The plates were then washed with deionized water and air-dried. Samples were stained with 100 μl of SRB solution (in 0.4% w/v in acetic acid) for 20 min at room temperature. The plates were then washed with acetic acid (1%) and air dried. Tris-base (10 mM, 100 μl, pH 10) was added to each well for solubilization. Optical density (O.D) values were measured at 540 nm with a reference wavelength of 630 nm using microtiter plate reader (VERSMax).

### Flow cytometry analysis of cell apoptosis and cell cycle

For cell apoptosis analysis, cells were harvested at 70∼80% confluence and incubated with reagent containing Annexin V-FITC and propidium iodide (BD Biosciences) for 15 min in darkness at room temperature. Apoptotic cells were analyzed using FACS Caliber flow cytometer (BD Biosciences).

For cell cycle analysis, cells were fixed in 70% ethanol at 4°C overnight and then treated with RNase A (50 μg/ml) and stained with propidium iodide (25 μg/ml) for 30 min at 37°C. Distribution of cell-cycle phases was determined using ModFit software (BD Biosciences).

### Cell migration and cell invasion assays

Cell invasive and migratory potentials were evaluated using a Transwell assay. Briefly, cell invasion assays were conducted using specialized MilliCell chambers (Millipore, Bedford, MA, USA). The inserts contained an 8 μm pore size polycarbonate membrane with a pre-coated thin layer of Matrigel (BD Biosciences). 10% FBS-containing medium was placed in the lower chambers to act as a chemo-attractant. Then, 1×10^5^ AsPC-1 and CFPAC-1 harvested from transfection in a 100 μl volume of serum-free medium were placed in the upper chambers and incubated at 37°C for less than 24 h. Invasive cells on the lower surface of the membrane, which had invaded the Matrigel and had migrated through the polycarbonate membrane, were stained by 0.1% Crystal Violet Staining Solution for 15 min. Cells on the upper surface of membrane were scraped off with cotton swabs and counted under a microscope in five randomly selected fields at a magnification of ×200 after dried. Migration assays were the same as above with the exception that no Matrigel was used and the permeating time for cells was less than 24 hours.

### RNA binding protein immunoprecipitation (RIP)

The RIP assay was performed using the Magna RIP™ RNA-Binding Protein Immunoprecipitation Kit (Millipore) according to the manufacturer's instructions. Briefly, cells were harvested and lysed in RIP Lysis Buffer. LncRNA MALAT-1 was immunoprecipitated with an EZH2 antibody. The magnetic bead bound complexes were immobilized with a magnet and unbound materials were washed off. Then, lncRNA MALAT-1 was extracted and analyzed by RT-qPCR. The primer pair of MALAT-1 for RIP assay was:

forward, 5′-GTGAGCAAACTGTGTTGGCGTG-3′,
reverse, 5′-CATCGAGGTGAGGGGTGAAGGG-3′.

### Chromatin immunoprecipitation (ChIP) assay

ChIP assays were performed on cell line DNA using an Imprint Chromatin Immunoprecipitation Kit according to the manufacturer's instructions (Millipore, USA). Briefly, AsPC-1 and CFPAC-1 cells (5 × 10^6^) were treated with 1% formaldehyde for 10 min for cross-linking, and then quenched by the addition of 0.125 M glycine. The cells were scraped with PBS solution and gathered after centrifugation at 800 g for 5 min at 4°C. Then, the cross-linked cells were re-suspended in SDS lysis buffer containing protease inhibitor cocktail II and the soluble chromatin was sheared to fragment DNA by nuclear lysis buffer. The fragmented chromatin samples were aliquoted as genomic input DNA or immunoprecipitated with EZH2 and H3K27me3 antibodies or IgG, and incubated at 4°C with rotation overnight. Immunocomplexes, collected by magnetic separator, were washed and eluted with ChIP elution buffer. DNA was purified on spin columns. The ChIP products and genomic input DNA were analyzed by real-time PCR with SYBR Green PCR Master Mix (Applied Biosystems, Foster City, CA). The three primer pairs of E-cadherin used for ChIP assays were:

forward, 5′-TGTCCGCCCCGACTTGTCTCTC-3′,
reverse, 5′-GTCCTCTGGCCCCAGCCTCTCT-3′;
forward, 5′-AGACCCCATCTCCAAAACGAACAAA-3′,
reverse, 5′-GCATAGACGCGGTGACCCTCTAGCC-3′;
forward, 5′-TGTCCGCCCCGACTTGTCTCTC-3′,
reverse, 5′-CGGTCCTCTGGCCCCAGCCTCT-3′.

### Statistical analyses

Data are presented as mean ± SD. Values and percentages between groups were compared using Student's t tests and chi-square tests, respectively. We analyzed the associations between the expression of EZH2 or E-cadherin and clinical characteristics with χ2 tests or Fisher's exact methods as appropriate. Overall survival (OS) was defined as the interval from date of diagnosis until death from any cause. The patients were censored if they were still alive or the patients lost to follow-up until the last follow-up. We assessed OS using a Kaplan-Meier method. Univariate and multivariate COX regression analysis was performed. Those parameters with a *P* value <0.05 in the univariate analyses were included in a Cox multivariate proportional hazards regression model. All *P* values were two sided, and the differences were considered significant at the value of *P* < 0.05. All statistical analyses were carried out using SPSS 17.0

## SUPPLEMENTARY FIGURES


