# Supplementary material for: EZH2 promotes cell migration and invasion but not alters cell proliferation by suppressing E-cadherin, partly through association with MALAT-1 in pancreatic cancer
Source: Oncotarget. 2016 Feb 3;7(10):11194–207. doi: 10.18632/oncotarget.7156 (PMC4905466; doi:10.18632/oncotarget.7156)
Supplement: Supplementary file 1 [file oncotarget-07-11194-s001.pdf]

SUPPLEMENTARY FIGURES

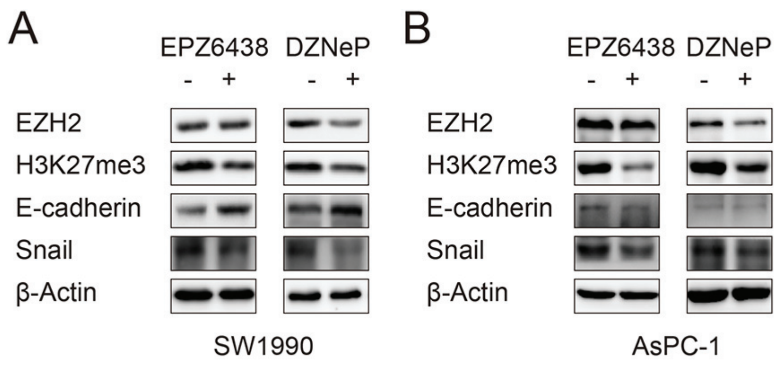

Supplementary Figure S1: The treatment of EPZ-6438 and DZNeP increased E-cadherin and decreased Snail and H3K27me3 expression in pancreatic cancer cells. A, B. The treatment of DZNeP and EPZ-6438 increased E-cadherin and decreased Snail, H3K27me3 expression in pancreatic cancer cell lines SW1990 (A), AsPC-1 (B).

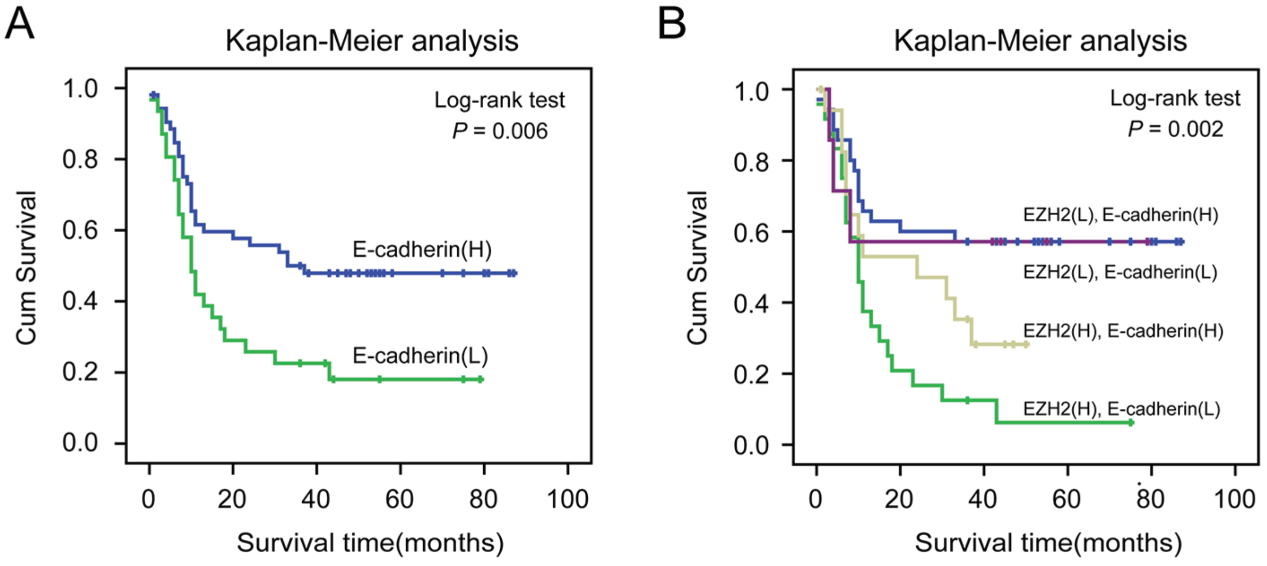

Supplementary Figure S2: Kaplan Meier analysis of correlation of EZH2 and E-cadherin protein expression with the prognosis of pancreatic cancer patients. A. E-cadherin expression is negatively correlated with advanced tumor stage. **B.** Patients with higher EZH2 expression and lower E-cadherin expression possessed the worst prognosis. The scales represent 50μm. “L” represents low, “H” represents high.

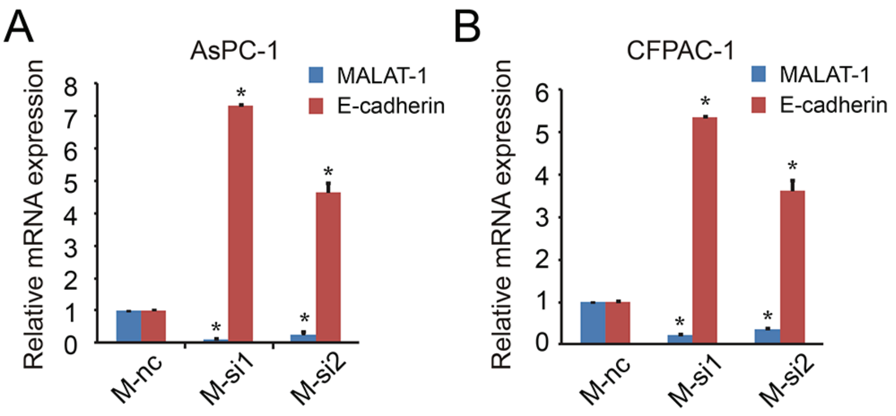

Supplementary Figure S3: The mRNA level of E-cadherin expression in MALAT-1-knockdown cells. A, B. The E-cadherin mRNA expression was increased after MALAT-1 knockdown in AsPC-1 (A) and CFPAC-1 (B) cells. "*" represent $P<0.05$ when compared with control group.

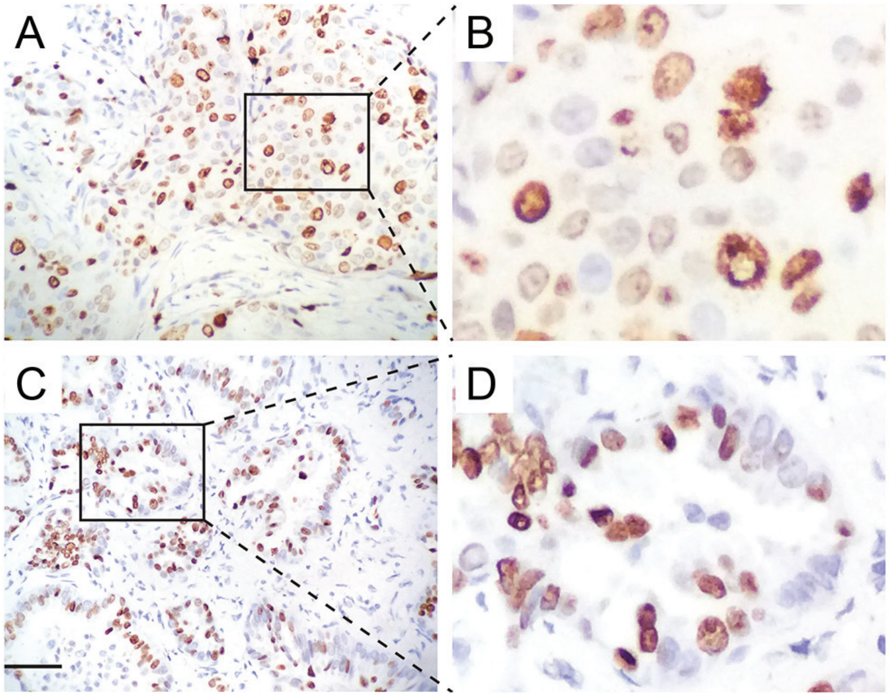

Supplementary Figure S4: Immunohistochemical staining for EZH2 expression in breast and lung cancer. Representative figures of EZH2 expression in breast cancer **A, B.** and lung cancer tissues **C, D.** The scales represent 50μm.
